# “It's Gonna Be a Stressful Day!”: How Stressor Forecasting Moderates Stress and Wellbeing in Real‐World Contexts

**DOI:** 10.1002/pchj.818

**Published:** 2025-02-24

**Authors:** Jamie S. Elsey, Sam Dutton, Monika Lohani

**Affiliations:** ^1^ Department of Educational Psychology University of Utah Salt Lake City Utah USA; ^2^ Department of Psychology University of Utah Salt Lake City Utah USA

**Keywords:** affective experiences, ecological momentary assessment, stress, stressor forecasting, wellbeing

## Abstract

Stressor forecasting predicts the likelihood of a stressful event occurring in an upcoming timeframe and can significantly influence everyday experiences. The current study aimed to understand how stressor forecasting may moderate links between hourly stress and affective experiences over the course of a day. An ecological momentary assessment approach was used to collect data from 304 participants about their predicted stressor forecasting and hourly stress to personally relevant stressors and affective experiences 10 times within a day. We predicted that stressor forecasting would moderate the relationship between stress and affective experiences (separately for negative and positive affect). Stressor forecasting significantly moderated the links between stress and negative affect, but not between stress and positive affect. These findings emphasize the meaningful implications that adverse stressor forecasting can have on daily wellbeing, which may lead to the development and maintenance of chronic stress.

## 1 Introduction

How does anticipating a stressful day predict one's experiences later that day? Past theoretical (e.g., Almeida 2005; Epel et al. 2018; Lazarus and Folkman 1984) and empirical (e.g., Almeida et al. 2020; Bolger et al. 1989; Charles et al. 2013; Neupert, Almeida, and Charles 2007; Piazza et al. 2013) research has well‐documented how experiences of daily stressors lead to increased daily negative affect (Almeida 2005; Bolger et al. 1989; Kramer et al. 2022; Stawski et al. 2008) and decreased positive affect (Lohani, Dutton, and Elsey 2022; Smyth et al. 1998). However, not all stressful events are random or unexpected; some stressors (such as an upcoming exam or deadline) can be anticipated, which may also cause experiences of stress. Recent research has started examining not only what happens after the occurrence of a stressor but also the process before a stressor occurs (Neupert et al. 2019) and how this anticipation can have downstream effects on everyday wellbeing (e.g., Kramer et al. 2022; Neubauer, Smyth, and Sliwinski 2018a, 2018b).

Stressor forecasting is the expectation of a stressful event in a defined timeframe (such as the upcoming day; Neupert et al. 2019). There is growing support for a recent perspective referred to as the conceptual framework of anticipatory stress and coping (Neupert and Bellingtier 2019) that theorizes that the anticipation of a stressful event has comparable consequences as the stress response following a stressor's occurrence, including psychological, physiological, health, and wellbeing impacts. A growing empirical literature has found support for this theoretical perspective by demonstrating that when forecasting stressors, individuals experience outcomes similar to the stress experienced post‐exposure, as evidenced by increased negative experiences, stress physiology, worse health, and cognitive deficits (Diachina and Neupert 2022; Neupert and Bellingtier 2019; Johnson and Neupert, 2023). For instance, similar to typical stress responses, anticipating and making predictions about a stressful event (e.g., daily election‐related stressors during an election year) are associated with increased negative affect (Kramer et al. 2019; Neubauer, Smyth, and Sliwinski 2018b; Scott et al. 2019; Smyth et al. 1998; Zhu and Neupert 2022). Anticipation of stress is also associated with increased salivary cortisol levels (Smyth et al. 1998; Kramer et al. 2019), deficits in cognitive functioning (e.g., working memory tasks; Hyun, Sliwinski, and Smyth 2019; Sliwinski et al. 2006), and poor physical health (Johnson and Neupert, 2023). Furthermore, anticipatory stress responses have been suggested to be an underlying mechanism that may lead to the development and maintenance of chronic stress (Scott et al. 2019; Smyth, Zawadzki, and Gerin 2013). As a result, stressor forecasting potentially moderates stress and wellbeing over the forthcoming period (e.g., minutes, hours, and days post‐anticipation). This hypothesis remains to be tested with frequent hourly assessments post‐forecasting, which was the focus of the current study.

Although research that examines the relationship between anticipatory stress and increased daily negative affect is growing, the research on stressor forecasting and positive affect is limited and inconsistent (Kramer et al. 2022; Neupert and Bellingtier 2019). At the same time, research on wellbeing has shown that it can be independently represented by negative as well as positive affect (Lucas, Diener, and Suh 1996). Indeed, previous work has called attention to the lack of research on positive affect's links with stressor forecasting (e.g., Neupert and Bellingtier 2019; Smyth et al. 1998; Kramer et al. 2022). Some work has found that anticipating stressful events is associated with lower positive affect (Smyth et al. 1998). In contrast, another study found that young adults (18–36 year‐olds) had an increase in positive affect when they forecasted specific daily stressors (Diachina and Neupert 2022). Therefore, there is mixed evidence and a limited understanding of stressor forecasting and its relationship to positive affect. A primary goal of this work is to address this knowledge gap. Because of the possible adverse chronic effects (Kramer et al. 2022; Scott et al. 2019; Smyth, Zawadzki, and Gerin 2013), it is critical to understand the potential influence of stressor forecasting on stress and affective experiences.

### 1.1 The Current Study

This project aimed to understand how stressor forecasting may have potential downstream effects on stress and wellbeing over the course of a day. Self‐reported negative and positive affect were assessed as a measure of daily wellbeing (Lucas, Diener, and Suh 1996). An important focus of this study was to examine the potential influence of stressor forecasting and affective experiences frequently because affect can change dynamically. Past research has found that emotional episodes change dynamically, and 80% of these episodes last about 1 h (Verduyn et al. 2009). Recent calls have recommended more frequent assessments to fully capture the variation in associations between stressor forecasting and affective wellbeing (Kramer et al. 2022; Neupert et al. 2019). Thus, to capture how individuals respond to personally relevant daily stressors, ecological momentary assessment (EMA) was adopted (Smyth et al. 2017). EMA methodology entails asking brief questions to capture psychological experiences (e.g., negative affect and stress) in responders' personally relevant and naturally occurring contexts that help improve ecological validity (for review, see Shiffman, Stone, and Hufford 2008). EMA often adopts repeated assessments over time (varying between minutes, hours, or weeks) depending on the frequency that is relevant to gain more reliable assessments by collecting data close to the psychological experiences. EMA enabled us to gather hourly dynamic changes in stress and affective experiences within the same day, which is a good temporal range to capture for affective constructs (Verduyn et al. 2009).

While previous work has examined the relationship between stressor forecasting and negative affect (Scott et al. 2019) and the relationship between stressor occurrences and negative affect (Neubauer, Smyth, and Sliwinski 2018b), limited work has examined stressor forecasting as a moderating link between stress level and negative affect. We examined the following two research questions. First, how does stressor forecasting moderate the association between hourly stress and negative affect? Based on previous findings with stressor anticipation and increased negative affect (Neubauer, Smyth, and Sliwinski 2018b; Scott et al. 2019; Smyth et al. 1998; Kramer et al. 2019), we hypothesized that higher levels of reported stressor forecasting would moderate the stronger positive association between stress level and negative affect. Second, how does stressor forecasting moderate the association between stress and positive affect? This was an exploratory research question owing to the mixed findings on anticipatory stress and positive affect (Diachina and Neupert 2022; Smyth et al. 1998).

## 2 Method

### 2.1 Participants

Three hundred four adults (*M*_age_ = 22, *SD* = 5.52, with 89.8% females, 9.4% males, 0.1% genderqueer, 0.6% selected other, and 0.3% chose not to disclose) participated in this study. The sample included 80.3% Caucasian, 7.9% Asian, 8% two or more races, 1.5% American Indian/Alaskan, 1.2% Black/African American, 0.9% other, and 0.3% other Pacific Islander. The highest level of completed education was 12.9% high school diploma, 1.1% GED, 18.2% college freshman, 20.7% college sophomore, 37.9% college junior, 6.4% bachelor's degree, 1.1% master's degree, and 0.4% J.D., M.D., or Ph.D. Participants were recruited from the university's research pool and received research credit for participating. All procedures were in line with and approved by the Institutional Review Board of University of Utah. Note that among the sample of 304, 30 participants did not report their stressor forecasting, which left 274 participants who did report stressor forecasting, even when they reported stress and affect during the day. These available data were utilized for planned analyses, that is, for the moderator analysis with stressor forecasting, data were only available from 274 participants.

### 2.2 Measures

#### 2.2.1 Stressor Forecasting

At the beginning of the day of the study, participants were asked to rate “Today: How frequently do you expect stressful events to occur?” on a five‐point scale from 0 (*not at all*) to 4 (*a great deal*). This question was on a Likert scale and adapted from past work on stressor forecasting (Scott et al. 2019; Neupert, and Bellingteir 2019).

#### 2.2.2 Stress

At each EMA event, participants were asked to indicate the level of stress they felt since the last sampling event, from 0 (*not at all*) to 4 (*a great deal*). Intraclass correlation (ICC) was calculated using an R package called performance in line with past longitudinal research to account for the nested nature of data when calculating the ICC for the model (Lüdecke et al. 2021; Nezlek 2012). The ICC for stress was 0.51.

#### 2.2.3 Negative and Positive Affect

Negative and positive affect were measured at each EMA event using a modified version of the differential emotion scale (Watson, Clark, and Tellegen 1988). Participants were presented with eight negative emotions (sadness, irritable, bored, anger, lonely, helpless, hopeless, and useless) and seven positive emotions (happiness, enthusiastic, love, proud, peace, purposeful, and amazement). For each word, participants were asked, “Since you last indicated, how much of the following do you feel.” Each word was rated from 0 (*not at all*) to 4 (*a great deal*). Negative and positive affect scores were calculated by averaging the emotional words' ratings, respectively. Following past work (Lüdecke et al. 2021; Nezlek 2012), a nested null model (with no predictors) was run to explain the composite affect score (separately for NA and PA), and the ICC for this model was calculated. The ICC was 0.70 for negative affect and 0.65 for positive affect.

### 2.3 Procedure

Participants completed the consent form after signing up for the study. They selected a day they would participate in the study. Accordingly, their phones were set up to receive text messages with links to the surveys using SurveySignal (Hofmann and Patel 2015). EMA was used to capture hourly changes in affective experiences throughout a typical weekday as participants went about their day in their naturally occurring contexts. A semi‐random beep design was utilized (Shiffman, Stone, and Hufford 2008), and surveys were sent at random times each hour between 10:00 a.m. and 8:00 p.m. A reminder message was sent after 15 min if participants did not open the link for a sampling event. The link expired half an hour after being first sent, so we could record responses temporally close to when the event sampling was randomly initiated. No other exclusion rules were applied to participants' data.

### 2.4 Compliance

Participants completed, on average, 8.49 out of 10 surveys; 40.5% completed all 10 surveys, 25% completed 9, 13% completed 8, 10.2% completed 7, 3.9% completed 6, and 7.4% completed 5 or fewer. 82.1% of participants completed the question “Today: How frequently do you expect stressful events to occur?”

### 2.5 Data Analysis Plan

All analyses were conducted using R Studio with primarily the following packages: psych, lme4, lmerTest, emmeans, performance, jtools and corrplot. A hierarchical linear modeling approach was adopted in line with past longitudinal EMA research (Lohani, Dutton, and Elsey 2022; Lohani et al. 2023). This allowed us to test our research question of interest regarding the role of stressor forecasting on the day's stress and affect links after accounting for the nested nature of the data. To address the aims of this study, multilevel models were employed with EMA events (Level 1) nested within individuals (Level 2). Time was included in the model to account for potential changes in affective experiences throughout the day. Participants were included as a random intercept. The effect of stressor forecasting and stress level on affective experiences was analyzed using equations 1 through 4. Person *j'*s affect at sampling event *i* (1–10) was predicted by the variables stress level (centered on participant mean) and time. Separate models were run to examine negative affect (Model 1) and positive affect (Model 2) as outcome variables.

Level 1 (event‐level):

(1) $${\text{Affect}}_{\mathrm{ij}}=\beta_{0j}+\beta_{1j}\left(\text{Stress Level}\right)+\beta_{2j}\left(\text{Time}\right)+r_{\mathrm{ij}}$$

On the person level, the intercept of affect was predicted by participants' stressor forecasting (centered on the grand mean). Similarly, the slope of stress was also predicted by stressor forecasting.

Level 2 (person‐level):

(2) $$\beta_{0j}=\gamma_{00}+\gamma_{01}{\left(\text{Stressor Forecasting}\right)}_{j}+\mu_{0j}$$

(3) $$\beta_{1j}=\gamma_{10}+\gamma_{01}{\left(\text{Stressor Forecasting}\right)}_{j}+\mu_{1j}$$

(4) $$\beta_{2j}=\gamma_{20}$$

Multilevel analyses were performed in R using the lme4 package (Bates et al. 2015).

To examine interaction terms, the association between stress level and affect was plotted at lower levels (−1 *SD*) and higher levels (+ 1 *SD*) than average levels of stressor forecasting. A simple slopes test was run to examine how the association between stress and negative affect varies across lower and higher levels of stressor forecasting. A planned post hoc comparison was run to examine the moderating effect of stressor forecasting at higher (+ 1 *SD*) and lower (−1 *SD*) stress levels on negative (Model 1) and positive (Model 2) affect.

## 3 Results

Table 1 shows the correlation coefficients for the variables of interest in this study after accounting for the nested nature of the data.

**TABLE 1:** Descriptive statistics and correlations for study variables.

Variable	*M*	*SD*	1	2	3	4
Negative affect	0.71	0.76	—			
Positive affect	1.38	0.88	−0.09***	—		
Stressor forecasting	2.04	0.89	0.22***	−0.09***	—	
Stress	1.94	1.21	0.52***	−0.13***	0.33***	—

*** *p* < 0.001.

### 3.1 Negative Affect

**FIGURE 1:**
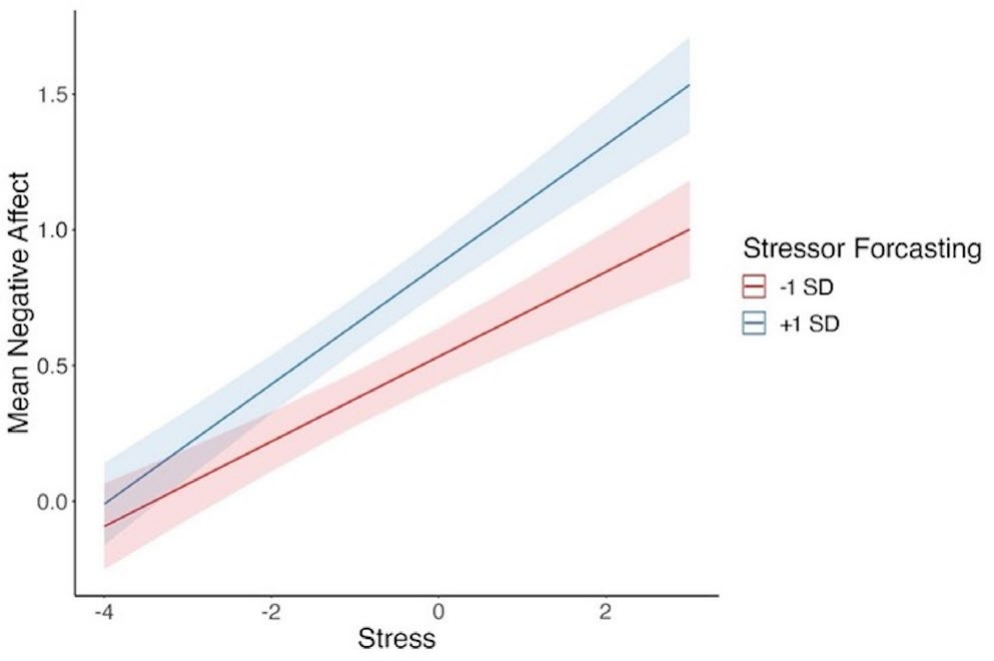
Stressor forecasting moderated the association between stress and negative affect. Stress is participant‐mean‐centered.

Descriptive statistics and correlations of the study variables are shown in Table 1 after accounting for the nested nature of data. Model 1 tested how negative affect was predicted by stress and time and moderated by stressor forecasting (see Table 2). Consistent with the hypothesis for question 1, stressor forecasting significantly moderated the links between stress and negative affect (*b* = 0.04, *SE* = 0.02, *p* = 0.02, 95% CI [0.01, 0.07]; see Figure 1). Time was a significant predictor of negative affect (*b* = −0.01, *SE* = 0.003, *p* = 0.005, 95% CI [−0.01, −0.001]).

**TABLE 2:** Multilevel regression results for negative affect.

	Model 1: Negative affect				
Predictors	Estimates	Std. error	*t*	df	*p*
Stress	0.19	0.01	13.67	258	< 0.001
Stressor forecasting	0.19	0.04	4.48	272	< 0.001
Time	−0.01	0.003	2.79	2133	= 0.005
Stress*stressor forecasting	0.04	0.02	2.36	247	= 0.019

*Note:* ICC = 0.75, marginal *R* ^2^ = 0.093, conditional *R* ^2^ = 0.77, stress was participant‐centered.

To examine stressor forecasting's moderating role in links between stress and negative affect, we found that for high‐stress levels, higher (than lower) forecasting was associated with higher negative affect, *t*(269) = 4.55, *p* < 0.0001, Cohen's *d* = 1.17. This suggests that stressor forecasting moderated the relationship between stress and negative affect. Additionally, examining the interaction effect at a higher stressor forecasting level (+ 1 *SD*), a comparison of higher levels (+ 1 *SD*) and lower levels (−1 SD) of stress revealed that participants who reported higher levels of stress had a significantly higher negative affect, *t* (199) = 11.61, *p* < 0.0001, Cohen's *d* = 1.5. Similarly, at a lower stressor forecasting (−1 *SD*), participants who reported higher (than lower) levels of stress had a significantly higher negative affect, *t* (250) = 7.87, *p* < 0.0001, Cohen's *d* = 1.06.

### 3.2 Positive Affect

**FIGURE 2:**
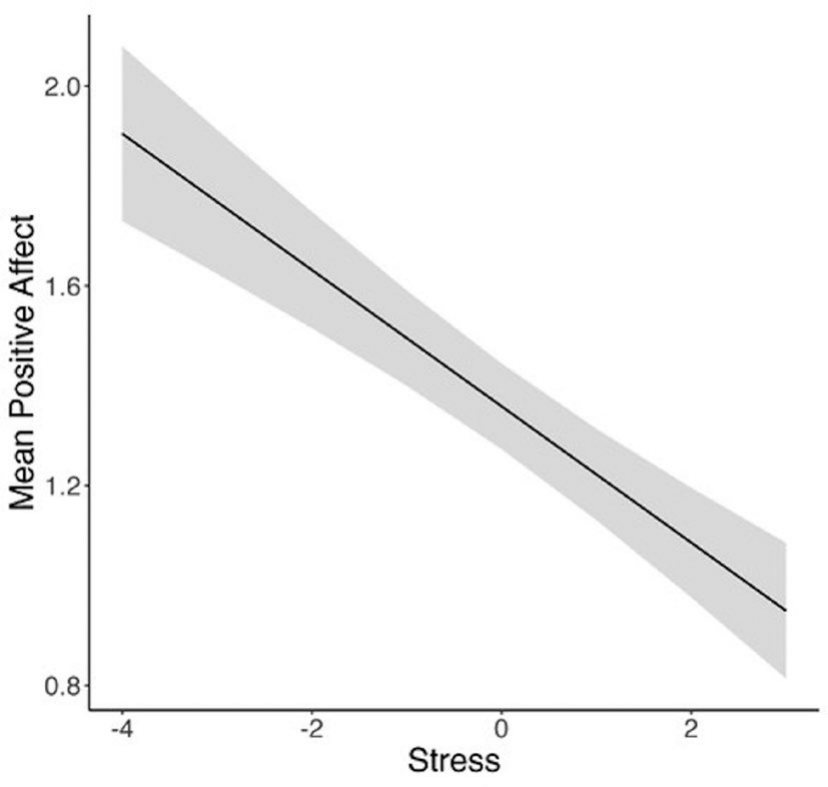
Higher levels of stress predicted lower positive affect. Stress is participant‐mean‐centered.

Model 2 tested how positive affect was predicted by stress and time and moderated by stressor forecasting (see Table 3). Stressor forecasting did not moderate the links between stress and positive affect (*b* = 0.0001, *SE* = 0.02, *p* = 0.998, 95% CI [−0.04, 0.04]). There was no significant effect of stressor forecasting on positive affect (*b* = −0.08, *SE* = 0.05, *p* = 0.09, 95% CI [−0.18, 0.01]). However, there was a significant effect of stress (*b* = −0.14, *SE* = 0.02, *p* < 0.001, 95% CI [−0.17, −0.10]; see Figure 2) on positive affect as well as time (*b* = −0.02, *SE* = 0.003, *p* < 0.001, 95% CI [−0.03, −0.02]).

**TABLE 3:** Multilevel regression results for positive affect.

Predictors	Model 2: Positive affect				
	Estimates	Std. error	*t*	df	*p*
Stress	−0.14	0.02	7.25	211	< 0.001
Stressor forecasting	−0.08	0.05	1.68	272	0.09
Time	−0.02	0.004	6.8	2132	< 0.001
Stress*stressor forecasting	< 0.01	0.02	0.003	202	0.998

*Note:* ICC = 0.7, marginal *R* ^2^ = 0.026, conditional *R* ^2^ = 0.707, stress was participant‐centered.

## 4 Discussion

Anticipation of stressors can represent an often overlooked pathway by which potential stressors can influence everyday stress and wellbeing. The current research adopted an EMA approach to examine how anticipation of stressful events during the upcoming day may potentially influence connections between hourly stress and affect experiences over the course of a day. When individuals had higher anticipation of stressors, a stronger association was found between stress and negative affect, but not for positive affect. These findings highlight the potentially deleterious effects that stressor forecasting can have on negative affective experiences, reflective of lower everyday wellbeing.

Even among instances when participants reported high stress, those who forecasted higher stress experienced higher negative affect. A possible explanation is that anticipating stressors and then actually experiencing them could feel worse, as reflected by higher negative affect. It could also be that the inability to regulate stress despite anticipating it may have contributed to higher negative affect, although this remains to be specifically tested. These findings extend previous work that has found that anticipating a stressor (Neubauer, Smyth, and Sliwinski 2018b) and the occurrence of a stressor (Scott et al. 2019) are associated with higher negative affect. Furthermore, these findings support the conceptual framework of anticipatory stress and coping (Neupert and Bellingtier 2019), which suggests that anticipation of stressors (before the occurrence of daily stressors) can impact experiences of daily stress and affect (in response to the occurrence of stressors). While previous work based on this framework has examined stressor forecasting over the course of a few hours and days (Neubauer, Smyth, and Sliwinski 2018b; Neupert and Bellingtier 2019; Scott et al. 2019), a novel contribution of this current work is that it investigated dynamic hourly stress and negative affect links within the same day for a more thorough understanding between these constructs of interest. The findings reveal that stressor forecasting may worsen the link between everyday stress and negative affect at an hourly timeframe and thus empirically supports the stressor forecasting framework (Neupert et al. 2019). Notably, in line with the conceptual framework of anticipatory stress and coping (Neupert and Bellingtier 2019), the current findings highlight the moderating role of high‐stressor forecasting in strengthening (and not weakening) the links between stress and average hourly wellbeing. This implies that mere anticipation may not be enough to counter the adverse effects of stress forecasting; instead, individuals may need help to be more proactive in addressing them.

Furthermore, the current findings highlight the importance of proactive coping efforts that can be implemented to manage upcoming stressors (Aspinwall and Taylor 1997; Neupert and Bellingtier 2019). Such proactive efforts could be helpful in mitigating the upcoming stressor or building more resilience towards it. It is likely that there is a lot of variability in affect regulation approaches individuals adopt in regulating their state when anticipating stressors (e.g., Author et al., under review). How to effectively manage the emotional toll of anticipated stressors remains an underexplored area of research. A challenge that remains in existing work is finding effective ways to separate proactive coping (that happens in response to anticipation of upcoming stressors) from reactive coping (that involves managing stressors after their occurrence). In future EMA research, questions will need to be carefully crafted to be able to distinguish coping efforts that are proactive versus reactive.

In contrast to negative affect, stressor forecasting did not explain the association between stress and positive affect. Current findings add to previously reported mixed findings with stressor forecasting and positive affect (Diachina and Neupert 2022; Smyth et al. 1998), and there are a few potential explanations for this finding. Participants may have employed regulatory techniques to deal with forecasted and unexpected stressors, such as emotion regulation (Lohani, Dutton, and Elsey 2022) or anticipatory coping (Neupert et al. 2019). For instance, past research has speculated that participants may have used anticipatory coping strategies, which may have impacted their reported positive affect (Kramer et al. 2022). On a related note, based on the stress‐buffering model (Pressman and Cohen 2005), positive affect may buffer the adverse effects of stress. Given the potential for positive affect to buffer stress (Pressman and Cohen 2005; Pressman, Jenkins, and Moskowitz 2019), examining how positive affect may influence stressor forecasting would be beneficial. For example, the effect of participants' positive affect at the time of stressor forecasting could be examined. Future work is needed to understand how stressor forecasting may lead to adopting anticipatory coping strategies to alleviate the adverse effects of anticipation on affective experiences.

### 4.1 Limitations and Future Directions

A few limitations and future directions need consideration. First, this work did not collect data on the type of stressors participants forecasted or whether those stressors actually occurred over the course of the day. Accuracy of stressor forecasting could be measured by asking participants to select the specific time frame when the forecasted stressor should occur and following up on whether the stressor happened in that time frame. There may be differences in accuracy forecasting between general and specific anticipated stressors. To examine these potential differences, participants could be asked to describe or select the type of stressor they forecasted and follow up on whether a general or specific stressor occurred during the timeframe. Second, anticipatory coping strategies were not examined in this work. It would be interesting to examine how coping strategies are implemented when anticipated stressors are experienced and how they affect emotional experiences. Furthermore, it would be helpful to separate the effect of the frequency of stressful events mixed with the intensity of the stressors, which could have distinct effects that remain underexplored.

Moreover, the stressors may be different depending on the day of the week, and future work should account for any additional variability this can contribute. Third, positive and negative affect (although it had a significant higher‐order interaction) decreased slightly over the day. This could be an effect stemming from natural changes in an emotional state approaching a more neutral affect over the course of a day that may or may not be reflective of EMA data collection. Future work is needed to separate the underlying explanation for changes over time. Fourth, the hourly semi‐random beep design was used to capture dynamic hourly changes in affective experiences and reduce recall bias (Shiffman, Stone, and Hufford 2008). While this design captures hourly changes, extending the time frame to include multiple days, weeks, or months of the semi‐random beep design would provide greater insight into the associations between stressor forecasting and affective experiences. Fourth, we had nearly 90% of females in our dataset, and in future work, a more balanced proportion of gender could allow for understanding gender differences.

## 5 Conclusion

Anticipation of a stressful day can have meaningful implications for the forthcoming stress experiences. The current findings suggest that stressor forecasting contributes to a heightened relationship between stress and negative affect later in the day. Thus, stressor forecasting has implications for everyday stress and wellbeing as it is a mechanism through which people experience pervasive negative affect and persistent chronic stress (Kramer et al. 2022; Scott et al. 2019; Smyth, Zawadzki, and Gerin 2013). Further work is needed to identify and mitigate the unforeseen adverse effects of stressor forecasting on daily health and wellbeing.

## Conflicts of Interest

The authors declare no conflicts of interest.

## Data Availability

The datasets generated during and/or analyzed during the current study are available from the corresponding author upon reasonable request.
